# Development and Feasibility of a Web-Based Decision Aid for Patients With Ulcerative Colitis: Qualitative Pilot Study

**DOI:** 10.2196/15946

**Published:** 2021-02-25

**Authors:** Andrew H Kim, Afaf Girgis, Peter De Cruz, Corey A Siegel, Neda Karimi, Sasha O Ruban, Alexandra J Sechi, Wa Sang Watson Ng, Jane M Andrews, Susan J Connor

**Affiliations:** 1 Ingham Institute for Applied Medical Research South Western Sydney Clinical School The University of New South Wales Sydney Australia; 2 Department of Gastroenterology Liverpool Hospital Sydney Australia; 3 Department of Gastroenterology The Austin Hospital Melbourne Australia; 4 Department of Medicine Austin Academic Centre University of Melbourne Melbourne Australia; 5 Section of Gastroenterology and Hepatology Dartmouth-Hitchcock Medical Center Lebanon, NH United States; 6 IBD Service Department of Gastroenterology & Hepatology Royal Adelaide Hospital Adelaide Australia; 7 Faculty of Medicine University of Adelaide Adelaide Australia

**Keywords:** shared decision making, decision aid, ulcerative colitis

## Abstract

**Background:**

Shared decision making (SDM) is becoming an important part of ulcerative colitis (UC) management because of the increasing complexity of available treatment choices and their trade-offs. The use of decision aids (DA) may be effective in increasing patients’ participation in UC management but their uptake has been limited due to high attrition rates and lack of a participatory approach to their design and implementation.

**Objective:**

The primary aim of this study is to explore the perspectives of Australian patients and their clinicians regarding the feasibility and acceptability of myAID, a web-based DA, in informing treatment decisions in UC. The secondary aim is to use the findings of this pilot study to inform the design of a cluster randomized clinical trial (CRCT) to assess the efficacy of the DA compared with usual care.

**Methods:**

myAID, a DA was designed and developed using a participatory approach by a multidisciplinary team of clinicians, patients, and nonmedical volunteers. A qualitative pilot study to evaluate the DA, involving patients with UC facing new treatment decisions and inflammatory bowel disease clinicians, was undertaken.

**Results:**

A total of 11 patients with UC and 15 clinicians provided feedback on myAID. Themes explored included the following: Acceptability and usability of myAID—myAID was found to be acceptable by the majority of clinicians as a tool to facilitate SDM, uptake was thought to vary depending on clinicians’ approaches to patient education and practice, potential to overcome time restrictions associated with outpatient clinics was identified, presentation of unbiased information enabling patients to digest information at their own pace was noted, and potential to provoke anxiety among patients with a new diagnosis or mild disease was raised; Perceived role and usefulness of myAID—discordance was observed between patients who prioritized voicing preferences and clinicians who prioritized treatment adherence, and myAID facilitated early discussion of medical versus surgical treatment options; Target population and timing of use—greatest benefit was perceived at the time of initiating or changing treatment and following commencement of immunosuppressive therapy; and Potential concerns and areas for improvement—some perceived that use of myAID may precipitate anxiety by increasing decisional conflict and impact the therapeutic relationship between patient and the clinician and may increase resource requirements.

**Conclusions:**

These preliminary findings suggest that patients and clinicians consider myAID as a feasible and acceptable tool to facilitate SDM for UC management. These pilot data have informed a participatory approach to the design of a CRCT, which will evaluate the clinical efficacy of myAID compared with usual care.

**Trial Registration:**

Australian New Zealand Clinical Trial Registry ACTRN12617001246370; http://anzctr.org.au/Trial/Registration/TrialReview.aspx?ACTRN=12617001246370.

## Introduction

### Background

Ulcerative colitis (UC) is an idiopathic chronic inflammatory condition involving the gastrointestinal tract, and it is characterized by relapsing and remitting symptoms of intestinal inflammation. Patients require long-term medical treatment, and in severe cases, they may require surgery to remove the entire colon. The trade-offs involving the available treatment options and their risk versus benefits are complex. Patients’ values and preferences heavily influence treatment choice and adherence [1]. Therefore, decision-making regarding therapeutics in UC encompasses not only the identification of the best treatment strategy for individual patients to maximize important outcomes such as quality of life and better disease control but also patient education, patient engagement, and effective communication to help promote adherence [2].

Shared decision making (SDM) is considered an important component of patient-centered care that enables and encourages patients to participate actively in the management of their health [3]. Such an approach has been found to result in better health outcomes and health care experience as well as increased treatment adherence [4]. The goal of SDM is that clinicians and patients will share their knowledge, values, and preferences and deliberate together on management decisions. Moreover, a recent survey suggests that most patients with UC wish to participate in SDM with their gastroenterologist when making treatment decisions [5].

Although SDM has a number of purported benefits, it requires a considerable amount of time and effort from the clinician to discuss the treatment options and explain their benefits and risks, while also helping the patient to identify which option best matches their preferences. With the increasing burden and incidence of UC, SDM is becoming an increasingly challenging task in a time-pressured, resource-limited clinic environment. Therefore, more effective ways of communication and patient engagement are needed.

Decision aids (DAs) are useful decision support interventions that may help to overcome the barriers to SDM by providing patients with easy-to-understand, evidence-based information about their available options outside the consultation timeframe, encouraging active engagement in the decision-making process and prompting patients to think through their values and preferences and consider them in making a decision [6]. The use of DAs in other chronic diseases has been associated with increased patient knowledge, less decisional conflict, and fewer patients remaining undecided or passive in the decision-making process [7]. DAs are available in various formats, ranging from simple pamphlets to elaborate videos, with web-based DAs becoming increasingly popular due to low production cost, ease of updates, and patient reliance on the internet for information [5,8].

Despite their potential to aid in participatory medicine, the application of DAs in UC management to date has been limited to decisions regarding surgical treatment [9,10], and available eHealth technologies incorporating self-monitoring and self-management functionalities have experienced high attrition rates, preventing their widespread uptake in clinical practice [11]. Importantly, many of these interventions have lacked a participatory health research design to maximize the participation of patients and clinicians in their design and development [12,13]. Furthermore, patient and clinician perspectives on the best approach to the use of these tools in routine clinical practice are currently poorly understood.

### Research Objectives

The primary objective of this study is to explore the perspectives of Australian patients and their clinicians regarding a web-based DA in informing treatment decisions in UC and investigate how such a tool may be best incorporated and used in clinical practice. The secondary objective is to use the findings of this pilot study to inform the design of a cluster randomized clinical trial (CRCT) aiming to assess the efficacy of myAID compared with usual care.

## Methods

### Development and Key Features of myAID

**Figure 1:**
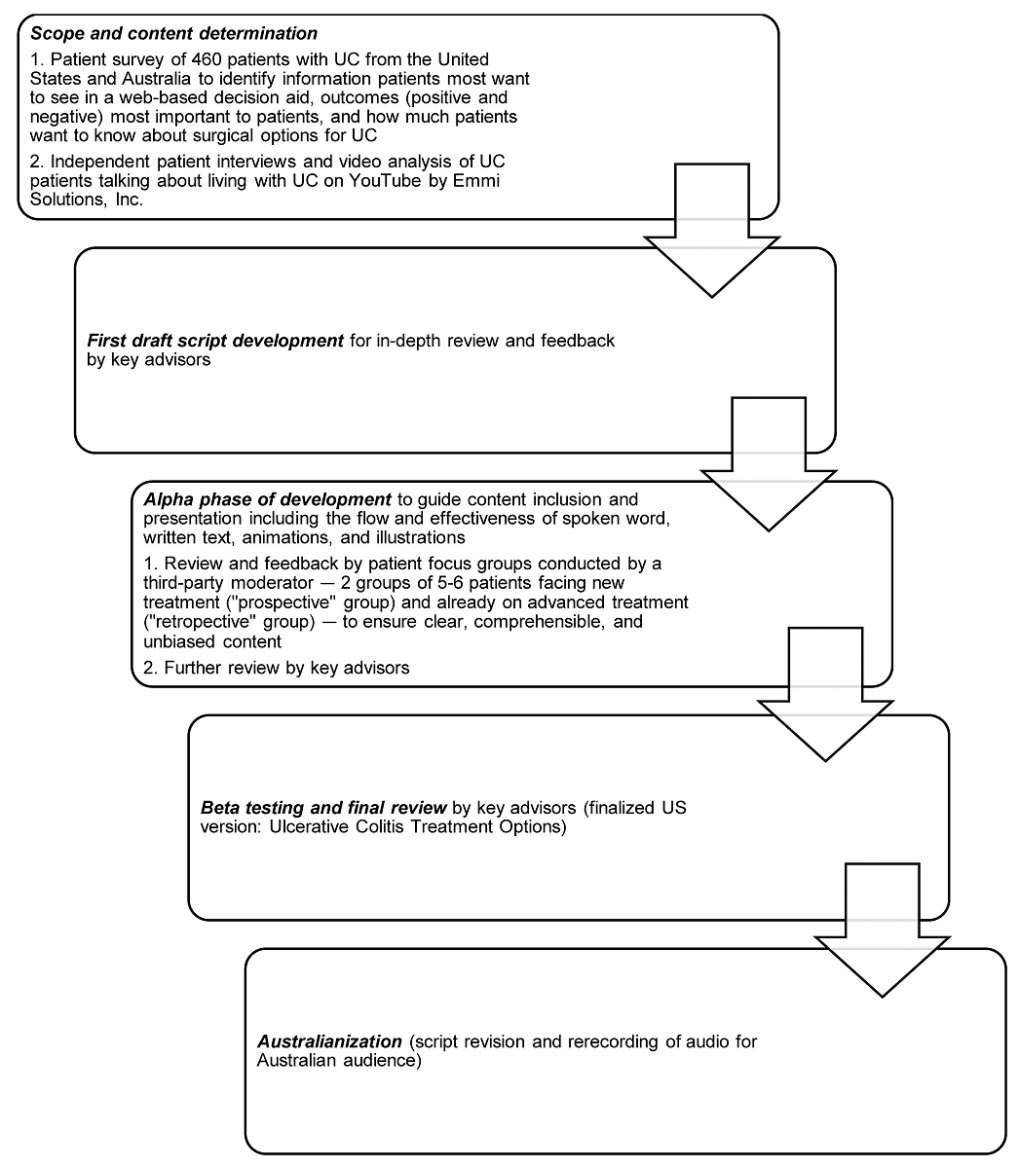
Development of the web-based decision aid.

myAID is a web-based multimedia DA. It is the Australianized version of the original US program named Ulcerative Colitis Treatment Options (Emmi Solutions, Chicago) designed to facilitate and support SDM for treatment decisions in UC management. Throughout its development and design, a participatory approach [12,13] was undertaken with direct input from patients and a multidisciplinary panel of clinical experts (inflammatory bowel disease [IBD] physicians, colorectal surgeons, and experts on SDM experienced with DA use; see *Acknowledgments* section). This process involved close reference to the International Patient Decision Aids Standards checklist [14] and a rigorous evaluation process including in-house and independent reviews (Figure 1) [15]. The web-based format was selected based on a previous survey involving patients with UC from both the United States and Australia [5]. The DA script and audio were *Australianized* (using Australian language and correct Australian medication names) through feedback from 1 patient, 4 nonmedical volunteers, 1 psychologist (AG), 3 Australian IBD physicians (SC, WN, and JA), and 2 Australian IBD nurses (As and ES), with rerecording of audio with the Australian script by Emmi Solutions in collaboration with Medibank Private Australia.

**Figure 2:**
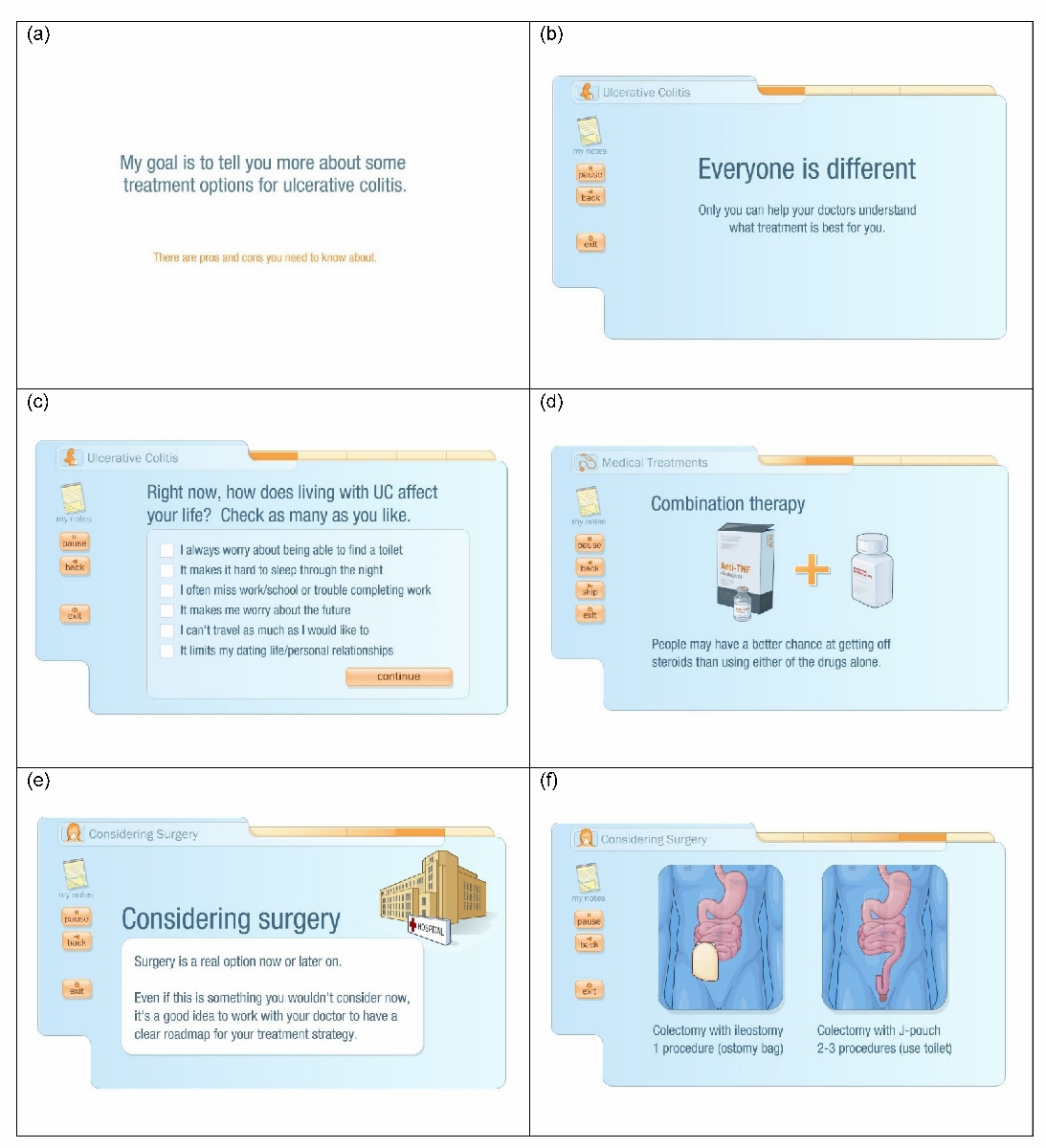
Sample screenshots of myAID.

myAID is web accessible (but not downloadable as a portable app) via a computer or a laptop with an internet connection, is available in English, and takes approximately 32 min if viewed uninterrupted. The content is organized into chapters (Textbox 1), which can be viewed as often as desired, and starts with information including common symptoms, treatment goals, and benefits and risks associated with well-controlled versus poorly controlled disease. It then presents a succinct summary of currently available medical and surgical treatments and their potential benefits and risks and a disclaimer confirming no support or influence from pharmaceutical companies. myAID has several interactive components that prompt patients to consider their personal treatment goals by asking questions about their current symptoms and concerns. They can select the specific details or information they wish to view about a particular aspect of treatment, such as information regarding surgery, including the details of the steps involved in creating a J-pouch or stoma and the potential complications such as pouchitis or peristomal hernia. Patients can take virtual notes during the video, print these along with a summary of the information presented in myAID, and return and skip to specific chapters after viewing the entire video. Refer to Figure 2 for sample myAID screenshots.

**Summary of myAID chapters.**

**Ulcerative colitis**

- An overview of ulcerative colitis, including anatomy, disease pathogenesis, typical symptoms, and treatment goals

**Medical treatments**

- An overview of available treatment options including brief discussions on the role of natural therapy including diet and probiotics as well as smoking

**Considering medications**

- Describes the importance of adherence and risk of flare when treatment is suddenly stopped, costs, and pros and cons of each medical (nonsurgical) treatment option including 5-aminosalicylates, steroids, thiopurines, and biologics (infliximab, adalimumab, golimumab, and vedolizumab)

**Considering surgery**

- Describes surgery as a treatment option including the reasons to consider surgery, benefits, what to expect after surgery, and the potential risks and also discusses ileostomy versus J-pouch formation

**Thinking it through**

- A review of treatment options covered in previous sections including how they are given, how quickly they work, efficacy, risk of infection and lymphoma, and ileostomy versus J-pouch

### Study Participants and Recruitment

#### Patients

Patients aged above 18 years with UC who needed to make a new treatment decision after a previous or current trial of 5-aminosalicylate (5-ASA) therapy were eligible to participate. Eligible patients were consecutively identified and referred to the research assistant (RA) by their clinicians during their outpatient clinic attendance at Liverpool Hospital, New South Wales (NSW). The RA provided patients with a participant information sheet, explained study participation, and answered any questions before obtaining written consent.

#### Clinicians

As this was a feasibility study informing the CRCT, only clinicians from sites that had been allocated to the CRCT intervention arm and had access to myAID were eligible to participate. Of the 25 eligible clinicians, 15 were randomly selected, and all the clinicians consented.

### Study Design

All consenting patients completed surveys to provide relevant sociodemographic and clinical information. Disease activity was determined by the Simple Clinical Colitis Activity Index (SCCAI) [16], where the score ranges from 0 to 19, with higher scores indicating greater disease severity and remission defined as a score ≤2. Patients viewed myAID in the clinic (using an on-site computer) or at home (on their personal computer) via a URL unique to each patient. Patients returned to their clinician within 2 weeks for a follow-up consultation to discuss and decide their UC management, after which they participated in a one-to-one structured telephone interview (Multimedia Appendix 1) with the RA to provide feedback about myAID. Interviews (approximately 20 min) were audio-recorded and transcribed verbatim for qualitative analysis.

Participating clinicians were provided with the myAID URL and contacted by the RA within 2 weeks to confirm that they had viewed myAID. A 20-min structured telephone interview was then scheduled with the RA or lead researcher (AK) to provide feedback about myAID; interviews were audio-recorded and transcribed verbatim for qualitative analysis.

Successive transcribed interviews were reviewed, and their content was analyzed independently by 2 researchers (AK and SR) using thematic analysis [17]. Both deductive and inductive approaches were used to identify, report, and categorize themes or patterns and to ensure saturation is achieved. Subsequently, the themes were further refined and reviewed to ensure reviewers’ agreement. Exemplary quotes were identified to denote each emerging theme during the analysis.

This study and the CRCT were approved by the Human Research Ethics Committee of South Western Sydney Local Health District and registered with the Australian New Zealand Clinical Trial Registry—ACTRN12617001246370.

## Results

### Participant Characteristics

A total of 11 patients were approached, and all of them were subsequently recruited (November 2015 to February 2016). The sample size was determined using the concept of thematic saturation. The majority of patients had left-sided disease (involvement limited to the proportion of the colon distal to the splenic flexure) for 2-10 years with a mean SCCAI of 6.5 (SD 5.6). A total of 15 clinicians with subspecialty interest in IBD participated (July to September 2016), the majority with ≥10 years of clinical experience and seeing ≥26 IBD patients per month. Tables 1 and 2 summarize the participant characteristics.

**Table 1:** Patient characteristics (N=11).

Characteristics		Total, n (%)	
**Age group (years)**			
	18-29		3 (27)
	30-39		4 (36)
	40-49		1 (9)
	50-59		3 (27)
**Gender**			
	Male		6 (55)
	Female		5 (45)
**Ethnicity**			
	White		5 (45)
	Other		6 (55)
**Education status**			
	Did not complete high school		2 (18)
	High school or equivalent		5 (45)
	Bachelor’s degree or higher		4 (36)
**Employment status**			
	Full-time		4 (36)
	Part-time		2 (18)
	Self-employed		2 (18)
	Homemaker		1 (9)
	Did not answer		1 (9)
	Disabled or unable to work		1 (9)
**Years since UC^a^ diagnosis**			
	<2 years		1 (9)
	2-10 years		10 (91)
**Disease extent**			
	Rectum only		1 (9)
	Left-sided		8 (73)
	Most or all of colon affected		2 (18)
**Previous treatment**			
	5-ASAs^b^		11 (100)
	Prednisone		7 (64)
	Thiopurines		3 (27)
	Infliximab		2 (18)

^a^UC: ulcerative colitis.

^b^5-ASA: 5-aminosalicylates.

**Table 2:** Clinician characteristics (N=15).

Characteristics		Total, n (%)	
**Age (years)**			
	30-39		6 (40)
	40-49		4 (27)
	50-59		5 (33)
**Gender**			
	Male		11 (73)
	Female		4 (27)
**Clinical setting**			
	Public only		1 (7)
	Private only		0 (0)
	Both		14 (93)
**Access to IBD^a^ nurse**			
	Full-time equivalent		11 (73)
	Part time		4 (27)
	None		0 (0)
**Clinical experience (years)**			
	<10		6 (40)
	10-20		5 (33)
	>20		4 (27)
**IBD patients seen per month**			
	≤25		1 (7)
	26-75		8 (53)
	≥76		6 (40)

^a^IBD: inflammatory bowel disease.

### Patient Interviews

Of the 11 patients, 10 provided feedback about the acceptability and usability of myAID, perceived role and usefulness of myAID, target population and timing and place of use, and potential concerns and areas for improvement (refer to Table 3 for subthemes and exemplar quotes).

**Table 3:** Patient feedback on acceptability of myAID.

Feedback^a^		Quote
**Acceptability and usability of myAID**		
	Easy to access, navigate, and use	“Everything was easy.” “I could go into more detail if I wanted to.”
	Information presented is adequate, well delivered, and unbiased	“It was spot on. It was perfect. I did not feel overwhelmed by it. But there was enough for me to take away and want to know more.”
	Content is informative and useful for patients	“Half the questions I was going to ask her were answered in that video. Helped me understand my situation and what the doctor was saying too.”
**Perceived role and usefulness of myAID**		
	Education (especially in terms of surgical treatment and combination therapy) and promoting adherence	“I had heard about things before and now I know what it is.” “now I am more aware of my options.” “I didn’t know about injections or surgery [before].” “I never took [the tablets] regularly. Now I will take them as doctor has prescribed.”
	Communication of concerns and participation in decision-making	“It did help me discuss my concerns and options with her [my doctor].” “Now I know what to talk about and what the doctor is saying and also my concerns.”
	Decision support	“It confirmed what I wanted to do and confirmed my decision. It was a big help.”
	Psychological support (reducing anxiety)	“I was scared to try other things before.” “I am more confident about my treatment.”
	Sharing information with family or other	“It can be explained to them in a way I wouldn’t be able to.”
**Suggested target population and timing or place of use**		
	Applicable at different stages of the treatment journey but may require caution in those newly diagnosed	“Overall the whole video was helpful it told you everything, all worst case scenario and everything [so] you know what is happening” “New people just diagnosed, [it] could scare them.”
**Suggested improvements and potential concerns**		
	Potential to increase anxiety and decisional conflict or burden—need for support from clinicians	“Like surgery and stuff it could be scary.” “None of them is what I want. When you only have so many options you have to take one and that’s the one I prefer more than the rest of them.” “...can be a bit of a shock too so maybe more help or support.”
	External factors may influence decisions—limitation of health care structure	“To show government we tried this option before we go to other option [of my choice].” (on Pharmaceutical Benefits Scheme restrictions)
	Suggestion for additional content	“It would be so good to know what to eat–do a video about that”
	Improved accessibility (on mobile devices)	“I wanted to do it on my mobile but I had to do it on my computer at home.”

^a^Themes arising from qualitative analysis.

#### Acceptability and Usability

Of the 11 patients, 8 viewed myAID in the clinic and 3 at home. Of the 10 interviewed patients, 9 watched myAID in one sitting, and all 10 patients found it easy to access myAID and navigate its contents and interactive components on the web-based platform; reported the amount of information presented as adequate and comprehensive; and found the information to be fairly presented (without bias), clear, and easy to understand. The majority of patients reported that myAID helped them with new knowledge about their disease and treatment options, leading to a much better understanding of the recent discussion with their clinicians. Many of these patients reported that their ability to contribute to the discussion was limited previously. The sense of control over the viewed content was received positively, and patients reported that this feature allowed them to digest potentially confronting information at their own pace.

#### Perceived Role and Usefulness

All patients considered myAID as a useful educational tool that helped to improve knowledge and understanding of UC and available treatment options as well as the rationale for maintenance treatment and adherence. myAID was perceived to improve their ability to communicate their concerns and participate in decision-making more effectively and confidently. Viewing myAID was believed to help most patients by reaffirming their treatment decision or helping them change it with increased confidence. Sections reported as particularly helpful were the discussion on surgical treatment and information regarding combination therapy (using an immunomodulator together with a biologic drug). The information presented and the sense of control it conveyed were reported to improve patients’ confidence and reduce their anxiety about new treatments. The majority of patients reported that they would share myAID with their family and relatives, explaining that they now had a way to explain their condition to a layperson in a language they can understand.

#### Target Population and Timing and Place of Use

Although myAID was considered acceptable and equally helpful for patients in various stages of UC in terms of disease duration and activity, some patients suggested caution against use in newly diagnosed patients for concerns of increasing anxiety, specifically referring to information about surgery. Several patients suggested that having their own time to digest the information away from the clinic was helpful. myAID was considered useful to view before consulting their clinician to understand their current situation and available treatment options and also afterward to help reaffirm decisions already made when introducing new treatment.

#### Potential Concerns and Areas for Improvement

Although patients indicated that myAID delivered new knowledge about their disease and available treatment options, they felt that information alone did not remove decisional conflict or burden and that they would like to follow this up with their clinician for support in the decision-making process. Inclusion of information about diet and enabling access to mobile devices were also desired by patients, the latter being thought of as a potentially more effective way of sharing information with others. One concern raised was that, in Australia, the Pharmaceutical Benefits Scheme influenced and limited treatment choices irrespective of patients’ and clinicians’ decisions (trial of thiopurines or methotrexate is mandated before funded biologic therapy for UC in Australia).

### Clinician Interviews

Subthemes and exemplar clinician quotes are provided in Table 4.

**Table 4:** Clinician feedback on acceptability of myAID.

Feedback^a^		Quote	
**Acceptability and usability of myAID from a clinician perspective**			
	Information is tailored for individual patients		“I can see benefit in people being able to go through this more than once, they could pick and choose what to view and not have to see what is not relevant anymore.” “You could hear it or not. I thought that was quite well done.”
	Information presented is adequate and delivered in a language that is suited to patients		“It was quite thorough without being over the top.” “Overall I really liked the non-confrontational way it described things and the way that it sort of went through educating people in language that is so accessible and non-threatening.”
	Content is useful and relevant for patients		“It covered a lot of very frequent questions that we get.” “The impression that I get is that it also draws on quite a lot of info from patients and their perspective.”
**Perceived role and usefulness of myAID from a clinician perspective**			
	Promoting treatment adherence		“...video is quite good at highlighting some of those points like ‘if you stop your medication it comes back’.”
	Patient education		“I thought it was done in a language that’s very easy for a patient to understand” “...they need time to digest information and then think about how they are going to use it”
	Potential to improve patient engagement and participation in decision-making		“We spend a lot of time talking in clinic and it would be nice to back it up and they can go through it in their own time.” “Sometimes it’s like we are putting them on the spot to make a decision (on the traditional approach to decision-making)”
**Suggested target population and timing or place of use**			
	Applicable at different stages of the treatment journey but particularly at the time of treatment escalation		“Depends on the course you are–at new diagnosis or 5 years down the track. Decision-making and info requirements are substantially different.” “Definitely early in their diagnosis and then the way it is structured it would be great at time of change of treatment or escalation.” “If someone presented with very severe disease you might want to show them that upfront because that would be incredibly helpful when you are scared and you don’t know what’s going on and you can see what options you have.”
	Early introduction may be beneficial but will require caution in those newly diagnosed		“I think so much depends on the level of education receives at the beginning and certainly time of diagnosis is a critical time.” “I don’t think at diagnosis because they are often overwhelmed with a lot of information.” “…people are often quite overwhelmed after diagnosis. So I would use it in first remission...and then each time there is a change or up titration.”
	Preferred setting for use is at home to allow for time and discussion but it could also be used in clinic		Home: “They need time to digest information and then think about how they are going to use it.” “I think the home is the best place so they can take their time and involve other people if they want.” Clinic: “One option to start it in clinic while they are waiting for us.”
	Support is needed to address questions promptly		“I think that people who have questions need to have them answered promptly.” “IBD nurse needs to be available otherwise questions will be forgotten about.”
**Suggested improvements and potential concerns**			
	Potential for information overload and intimidation—need for careful selection of patients		“It would be quite an intimidating thing for new patients to be confronted with things like surgery.” “Not having to hear about detail about surgery that they might not want to hear from a computer for the first time.”
	Potential for decisional conflict and disagreement		“Patients feeling like they have to decide. If they come to me and say I want this and if I don’t think that’s right it could create a problem.”
	Potential for outdated information		“Challenges are always to keep info up to date as we get new drug approvals.”
	Potential for increased resource use		“Making sure that there is a short time between them viewing it and coming back...if you leave it too long then the opportunity is lost...I don’t think it should be more than a couple of weeks.”
	Suggestion for additional content, features, and structural elements; reference to patient support programs; improved navigation and ability to print; and improved access		“Challenges are always to keep info up to date as we get new drug approvals.” “...have a scoring system so when they come back to clinic after seeing it, they have a validated index to then discuss. Could be clinical activity index or a PRO (patient reported outcome).” “Link to patient support programs.” “they could print something out at the end so they could discuss it with their doctors.” “Patients are always on their mobiles. It’s easier to have time. Needs to be mobile able.”

^a^Themes arising from qualitative analysis.

#### Acceptability and Usability

**Figure 3:**
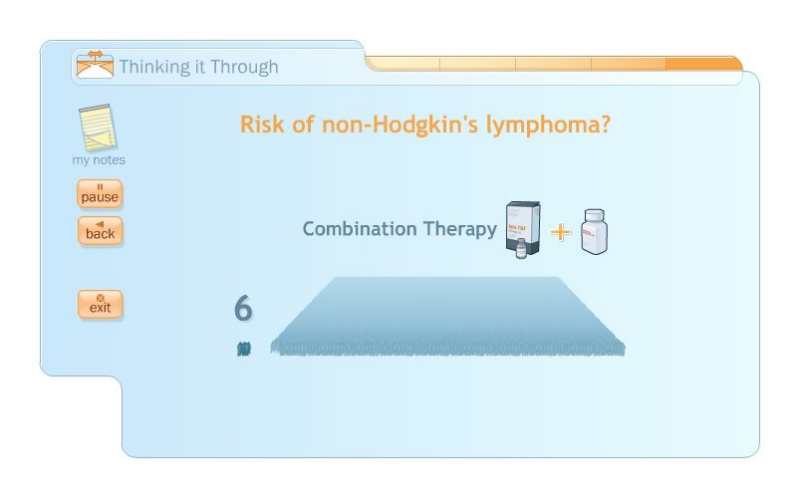
Visual representation of risk of lymphoma (sample screenshot from myAID).

myAID content was considered to be clear and well presented; the amounts of information and language were deemed appropriate; and visual presentation of risk, such as for lymphoma (Figure 3), was considered particularly useful. Information presentation and the ability to tailor viewed content were considered to be well done; no major content modifications were recommended. The majority of the 15 clinicians (n=11) indicated that they would readily welcome myAID as a *positive addition* to their practice, 2 indicated that they would potentially use it, and the remaining 2 were uncertain. Those in favor of including myAID in their practice suggested that it could (1) improve time efficiency, as a result of patients continuing with the education in their own time; (2) deliver reliable information, which clinicians agree is accurate and evidence-based; (3) improve communication and therapeutic relationships; and (4) facilitate greater patient engagement and participation in SDM, potentially improving treatment adherence. Potential reasons for not routinely using myAID included (1) uncertainty of its benefits over carefully conducted face-to-face consultations, for example, for long-term patients with complex disease and treatment history; (2) feeling of *imposing* decisions on patients with potential for unnecessary anxiety or patient-clinician conflict; and (3) potential for increased resource and communication needs as a result of questions arising from using myAID.

Some clinicians suggested that there would be a potential for the DA to result in disagreement between the patient and clinician, which might impact the therapeutic relationship. In particular, it was expressed by some clinicians that the DA may provide patients with too much autonomy regarding their treatment decisions, the complexity of which might be better directed by their clinician, particularly in the setting of the software providing more generic and less tailored information to an individual patient’s need.

#### Perceived Role and Usefulness

Clinicians perceived that myAID would be useful as a long-term educational tool to supplement clinic visits and support decision-making for patients throughout their disease course. Clinicians suggested that one of the primary functions of DAs such as myAID would be to emphasize the importance of treatment adherence and the risk of the disease itself, and additionally, clinicians wanted to see further functions included that will enable monitoring and tracking of patient clinical information such as disease activity.

#### Target Population and Timing and Place of Use

Although all clinicians suggested that myAID, if used, should be used early in the disease course, it was highlighted that individual patients have differing information needs based on diagnosis recency, disease behavior, education, treatment history, and patient-clinician relationship. The majority of clinicians considered myAID to be of greatest benefit to patients at times of treatment escalation, specifically when considering treatments beyond 5-ASAs when trade-offs become more complex. Half the clinicians also considered use at diagnosis, although the remainder were concerned that this timing would provide patients with too much information, causing more anxiety than necessary, particularly if their disease was mild. There were differing views about its use in acutely unwell patients in the hospital setting among clinicians, some reporting potential utility as an educational tool providing a broad overview of all available treatment options for these patients, whereas others felt that it was less appropriate given the presence of other factors influencing treatment decisions and the time pressures in this context. Some clinicians felt that it was important that information about surgery was provided by clinicians initially. All clinicians felt that patients should access myAID in the privacy of their home, although some also perceived it to be beneficial to view it in the clinic before their consultation. It was universally agreed that myAID should be made accessible on mobile devices, particularly for patients with active disease and for patients to be reviewed either in the clinic or via phone consultation (gastroenterologist or IBD nurse) within 2 weeks of viewing myAID to facilitate decision-making and address questions promptly. However, all of the clinicians also highlighted the difficulty in scheduling such a visit and stated the importance of nursing follow-up support.

#### Potential Concerns and Areas for Improvement

In addition to issues already highlighted, clinicians identified the need to regularly update content (new information and drugs), citing the imminent arrival of biosimilars in Australia and potential future therapies including fecal microbiota transplantation. Further tailoring of information specific to the Australian environment was also suggested, such as greater emphasis on the risk of skin cancer for patients considering thiopurines. Additional support for patients, such as inclusion of links to patient support programs or organizations (eg, Crohn’s and Colitis Australia), was also suggested. Although not identified as major issues, further improvements thought to be important included improved navigation between chapters (to allow for more rapid access to information of interest), enabling access in areas without internet connection, and ability to selectively print information included in myAID.

## Discussion

### Principal Findings

In this study, we have demonstrated that myAID, a web-based DA, is an acceptable and useful tool that can be used to facilitate SDM regarding medical and surgical treatments among patients with UC. Furthermore, using a participatory approach [13] in this pilot study, we obtained feedback from patients and clinicians that will enable us to optimize its usefulness and refine the clinical trial design used to evaluate its effectiveness.

The acceptability of DAs to facilitate SDM has been variable, and attrition rates associated with eHealth interventions have been high [12]. In our study, the majority of clinicians welcomed the prospect of SDM and use of the DA as an educational tool at times of treatment escalation, particularly when commencing immunosuppressive therapy to provide decision support and promote adherence. However, there were individual clinicians whose level of comfort using myAID varied depending on the clinical scenario to which it would be applied. Some clinicians did not perceive there to be any benefit from the DA over face-to-face consultations and suggested that the DA may be of limited value among patients with complex chronic disease, reinforcing the findings of Siegel et al [8] who found similar views expressed by gastroenterologists in relation to SDM. Moreover, some clinicians expressed greater risk of decisional conflict if patients felt obliged to make decisions using the DA, where previously their decisions would have been guided by the clinician, particularly at the time of diagnosis. These observations highlight the variation that is often observed in clinicians’ approaches to patient education and practices. Although DAs have the potential to improve the quality of health care delivery, by minimizing variation in care, their capacity to do so is only as effective as their uptake by patients and their clinicians [18].

Patients often find the time restrictions and busyness of the outpatient clinic a difficult environment to obtain adequate information about their disease and treatment options, which often limits their ability to participate in SDM. In this study, the DA was accepted as a tool to supplement the provision of information by the majority of clinicians and valued by patients as an opportunity to engage in SDM. Although the majority of patients and clinicians found the content acceptable, there was a minority who expressed that the DA may provoke unnecessary anxiety, particularly among patients with a new diagnosis or mild disease. In particular, it was felt by some that DAs may be less appropriate for newly diagnosed patients who might benefit from a more tailored approach to patient education. Patients expressed satisfaction with the unbiased presentation of information and sense of control they had over the range and depth of information provided, which appeared to increase their sense of autonomy and enabled them to digest potentially confronting information at their own pace.

Previous studies have suggested that there is often discordance between what patients and clinicians prioritize and find useful in eHealth tools [8,11]. In this study, we identified differences between patients’ and clinicians’ perceptions regarding their role in management. In particular, patients expressed a desire to voice their preferences about treatment decisions, whereas clinicians perceived that one of the primary roles of myAID was to emphasize the importance of treatment adherence. This is in agreement with previous studies on eHealth technologies that have suggested that patients tend to prioritize convenience in contrast to clinicians who prioritize adherence [13]. Nonadherence remains a key barrier to the efficacy of medical treatment in UC, with rates up to 70% with 5-ASAs [19]; it has emerged that nonadherence does not relate to forgetting to take medication alone but may be voluntary, which may be attributed to patients’ lack of belief in the value of their medical therapies [20,21]. This pilot study showed that myAID has the potential to converge patient and clinician perspectives by helping patients better understand the rationale for maintenance treatment and adherence and allowing for discussion of specific concerns or preferences. Existing literature has also previously suggested that DAs can reduce the discordance between patient and physician priorities [7].

Patients tend to overestimate the benefits of treatment and underestimate their harm [22]. SDM has been suggested as a strategy that may overcome this mismatch between patients’ and clinicians’ expectations [3]. In this study, information provided on surgical treatment and combination therapy was considered to be the most useful function of myAID by patients. In particular, patients expressed that the DA prompted them to openly discuss surgery with their clinicians and explore their values and preferences. Discussion about surgery is often neglected in routine UC management [23]. Although a DA has previously been developed for patients with UC to facilitate surgical decision-making between an end-ileostomy and ileal-pouch anal anastomosis [10], our DA was designed to place surgery in the context of medical treatment options at an earlier juncture in a patient’s disease course. Early discussion of medical versus surgical treatment options facilitated by the DA serves the purpose of informing and reminding patients and their clinicians about the range of treatment options available, together with their associated risks versus benefits, which might help align patients’ and clinicians’ expectations.

### Alterations in Study Design for the Planned CRCT

On the basis of the feedback obtained from this pilot study, specific alterations made to the study design for the planned CRCT included the exclusion of patients with newly diagnosed UC and those with acute severe UC requiring inpatient treatment. Drivers for these changes included concerns over heightened anxiety levels at the time of a new diagnosis and in the setting of an inpatient flare of disease, together with the time pressures associated with consultations and decision-making for both newly diagnosed and acutely unwell patients. The DA was considered to be more suitable for patients who were about to embark on immunosuppressive therapy, biologic therapy, or surgery, as an educational platform to help them consider the risk versus benefit of such management strategies. Given the preference expressed by patients to use the DA within the comfort of their own home, the study methods for the proposed CRCT were altered to provide all patients with a URL to allow them to use the DA from home. A scheduled study visit to follow-up within 2 weeks after the initial use of myAID was also introduced to help answer any questions or resolve any decisional conflict. Further amendments to the content and structure of the DA will be considered based on the suggestions received in this pilot study and feedback resulting from the CRCT.

### Limitations

To our knowledge, there are a number of DAs that have been developed to facilitate SDM for UC, each of which have different functions [12]. However, there is limited guidance as to what constitutes a good DA, as there are no universal measures available to determine optimal function except for one consensus checklist that documents quality criteria for development [14]. Although some DAs are designed to facilitate self-management via symptom monitoring and decision support [24,25], myAID’s focus is on education as a strategy to increase patient participation in their treatment choices in an effort to increase patient engagement in their health care. Although we acknowledge that a limitation of our pilot study was that the sample size was small, the qualitative nature of our study is unique in that it represents one of the few studies that has adopted a participatory approach, involving both patients and clinicians, in the design and development of the DA as well as the design of the planned CRCT to evaluate its effectiveness. We further acknowledge that a limitation of myAID is that it is currently only available as a web-based medium via computer owing to its interactive component requiring the use of Flash media. However, although patients expressed a preference for mobile access to the DA, its desktop application was not identified as a major issue by the pilot study patients. The need for web literacy and English and Spanish language comprehension (in the Australian and US versions, respectively) are also limitations of the DA. However, these limitations are not felt to be insurmountable, as the platform used to create the DA can be customized for mobile device use and programmed for several other languages.

### Conclusions and Future Directions—Unanswered Questions

The findings of this pilot study suggest that a web-based DA is an acceptable and useful tool to support decision-making regarding medical and surgical treatments among patients with UC. Using a participatory approach to engage patient and clinician feedback, the study design for the planned CRCT has been refined, thereby increasing the likelihood of being able to accurately evaluate whether myAID offers any benefit over usual care. Whether the use of DAs such as myAID that promote SDM will translate into clinically meaningful outcomes for patients remains to be seen and is the subject of our planned national CRCT.

## Appendix

Multimedia Appendix 1 Interview guide for patients and clinicians.

## Abbreviations

5-ASA: 5-aminosalicylates
CRCT: cluster randomized controlled trial
DA: decision aid
IBD: inflammatory bowel disease
NSW: New South Wales
RA: research assistant
SCCAI: Simple Clinical Colitis Activity Index
SDM: shared decision making
UC: ulcerative colitis
