# Supplementary material for: Development and Feasibility of a Web-Based Decision Aid for Patients With Ulcerative Colitis: Qualitative Pilot Study
Source: J Med Internet Res. 2021 Feb 25;23(2):e15946. doi: 10.2196/15946 (PMC7952232; doi:10.2196/15946)
Supplement: Multimedia Appendix 1 [file jmir_v23i2e15946_app1.docx]

**APPENDIX 1. Interview Guide for Patients and Clinicians Interviews.**

*Patients:*

- Did you have any difficulty accessing or viewing the decision aid? (If so, what were they? How would you suggest we improve this for other patients?)
- Did you view the entire decision aid video? (If not, why not?) Did you view the video in one sitting? (If no, why not?)
- How would you rate the amount of information in the decision aid? How clear was the information?
- Do you think each of the treatment options were presented to you fairly for you to make an informed decision?
- Did the decision aid increase your understanding about UC and the available treatments for UC? Please explain. Which part of the decision aid do you think is the most helpful?
- Did you feel that the decision aid has helped you communicate your concerns with your doctor?
- Did you/will you share the decision aid with family members or friends? (Do you think it would be useful to share the decision aid with family/friends?)
- Which treatment for UC have you decided to use?
- After viewing the decision aid, did you change your mind about your treatment?
- Is your plan for treatment the treatment that you want?

*Clinicians:*

Need to address:

1. Are you as a clinician interested in using the decision aid?
2. When in the patient journey should the patient view the decision aid?
3. When should the patient view the decision aid?
4. When and how should the follow up decision consult take place?

- What did you most like about the decision aid?
- What did you least like about the decision aid?
- What are your views about the best place for a patient to view the decision aid? (home or clinic)
- When in the patient journey do you feel it would be most effective for the patient to view the decision aid? e.g. at diagnosis or at the time of change of treatment or would you use it at multiple points in the patient journey?
- What would be the most feasible timeframe for the patient viewing the decision aid and having a follow up consult with you to make a decision about therapy?
- Is the decision aid a tool which you would implement in your clinical practice? Why or why not?
- Is there anything else we could do to make it more useful for patients?
- Proposed RCT study flow is as follows: patient has a consult with the clinician at which time they will either be diagnosed with UC or have failed their current treatment – patient views the decision aid at home – then has a follow up (decision consult) with their clinician within two weeks of their initial consult. Do you think this is feasible for your practice? If not, how can we improve the flow?
